# Methanol contamination in traditionally fermented alcoholic beverages: the microbial dimension

**DOI:** 10.1186/s40064-016-3303-1

**Published:** 2016-09-20

**Authors:** Elijah Ige Ohimain

**Affiliations:** Ecotoxicology Research Group, Biological Sciences Department, Niger Delta University Wilberforce Island, Amassoma, Bayelsa State Nigeria

**Keywords:** Traditional fermentation, Indigenous fermentation, Pectin, Pectin methyl esterase, Toxic ethanol, Methanol, Volatile congeners, Raffia palm

## Abstract

Incidence of methanol contamination of traditionally fermented beverages is increasing globally resulting in the death of several persons. The source of methanol contamination has not been clearly established in most countries. While there were speculations that unscrupulous vendors might have deliberately spiked the beverages with methanol, it is more likely that the methanol might have been produced by contaminating microbes during traditional ethanol fermentation, which is often inoculated spontaneously by mixed microbes, with a potential to produce mixed alcohols. Methanol production in traditionally fermented beverages can be linked to the activities of pectinase producing yeast, fungi and bacteria. This study assessed some traditional fermented beverages and found that some beverages are prone to methanol contamination including cachaca, cholai, agave, arak, plum and grape wines. Possible microbial role in the production of methanol and other volatile congeners in these fermented beverages were discussed. The study concluded by suggesting that contaminated alcoholic beverages be converted for fuel use rather than out rightly banning the age—long traditional alcohol fermentation.

## Background

Beverage ethanol production via fermentation is an age long tradition in many parts of the world. In the tropical world and elsewhere, indigenous people are involved in the entire value chain of traditional alcohol fermentation. Jespersen (2003) reported that most beverages and foods in Africa are produced at household level or on small industrial scale often of varying qualities. Aiyeloga et al. (2014) reported the potentials of raffia palm wine in sustaining livelihood in rural and urban populations in Nigeria. However, in Africa, Asia and South America, there has been an increasing incidence of methanol contamination in traditionally fermented alcoholic drinks (WHO 2014). Several cases of methanol poisoning have been reported in India and elsewhere. For instance in 2008, over 180 persons were killed in Bangalore and in 2009, 138 were killed in Gujarat, India. In 2015, 27 persons died in India after consuming toxic ethanol. In 2009, 25 persons died in Indonesia after consuming fermented palm wine containing methanol. About 130 persons died in some India villages in 2011 linked to poisonous ethanol consumption. In Czech Republic, 127 persons were poisoned from contaminated alcohol, out of which 42 died (Vaskova 2013). In 2014, the World Health Organization (WHO) alerted that there have been increasing outbreaks of methanol poisoning in several countries including Kenya, Gambia, Libya, Uganda, India, Ecuador, Indonesia, Nicaragina, Pakistan, Turkey, Czech Republic, Estonia and Norway. The size of these outbreaks ranged from 20 to over 800 victims, with case fatality rates of over 30 % in some cases (WHO 2014). Lachenmeier et al. (2011) evaluated the risk of contaminated unregulated alcohol in the European Union.

In Nigeria, between April and June 2015, a total of 89 persons died following the consumption of locally produced ethanol beverage called kaikai/ogogoro/apeteshi or illicit gin. Kaikai is produced mostly from the sap of raffia palm and oil palm and to a lesser extent from other palms such as date palm, nipa palm etc. Laboratory analysis carried out by WHO and NAFDAC (National Agency for Food, Drug Administration and Control) show that the beverage contain 16.3 % methanol, while the blood methanol concentration of victims was found to be 1500–2000 mg/l. Victims exhibited symptoms of methanol poisoning including loss of consciousness, dizziness, weakness and breathing difficulties, blurred vision and blindness, weight loss, headache, abdominal pains, nausea, diarrhea and vomiting (Methanol Institute 2013). WHO (2014) reported that blood methanol concentration above 500 mg/l is associated with severe toxicity, whereas concentration above 1500–2000 mg/l causes death in untreated victims. While investigation is ongoing on the source/origin of methanol in the beverage, the Federal Government of Nigeria (FGN) placed a ban on the production, sale, distribution and consumption of locally fermented beverage in Nigeria. Enforcement of the ban was heightened in the months (June–August 2015) following the incidence, but as of the time of writing (November 2015) enforcement has slacked. But the ban on the age long fermentation processes could have major impacts on the local economy. For instance, over 50 million people consume palm wine in Southern Nigeria (Obahiagbon 2009).

Raffia palm, which is among the most diverse and geographically widespread palm, is found in Africa, Asia and South America (Oduah and Ohimain 2015). The palm has many potential uses (Oduah and Ohimain 2015) but it is currently undertilized (Ohimain et al. 2015). Production of beverage ethanol from raffia palm provide a source of employment especially for rural people (Obahiagbon and Osagie 2007; Ohimain et al. 2012). Aiyeloja et al. (2014) studied the potential of raffia palm in the sustenance of rural and urban population in Nigeria. They found that raffia palm beverage value chain provides profit of ₦50,000–₦90,000 ($ 1 = ₦220) to producers and ₦45,000–₦70,000 to marketers. The complete ban on traditionally fermented beverages could be detrimental to the country’s economy especially at a time when most economics are under recession, with high inflation and un-employment rates. Nigeria is currently experiencing an economic downturn due to low crude oil prices. Hence, there is the need to establish the source/cause of methanol in traditionally fermented alcoholic beverages. Methanol Institute (2013) reported that methanol is often deliberately added to alcoholic beverages by unscrupulous and illegal criminal enterprises as a cheaper alternative to the production of cheaper ethanol. This may be unlikely in Nigeria and many other developing countries where methanol is not domestically produced but imported at costs higher than the cost of alcoholic beverage. For instance, domestically produced ethanol (40–60 % alcohol content) is quite cheap costing ₦20 per shot of 30 ml i.e. ₦670/l as against ₦5168/l of 99.85 % methanol (excluding importation and duty costs). Hence, there is need for research to focus on other possible sources of methanol in locally fermented beverages. WHO (2014) reported that outbreaks of methanol often occur when methanol is added to alcoholic beverages. Ohimain et al. (2012) reported that alcoholic beverages are produced in Nigeria using rudimentary equipment under spontaneous fermentation, which lacked effective controls and are carried out by uneducated rural workers with poor hygiene in an unsterile environment. Traditional fermentation is carried out by mixed cultures consisting of yeast, other fungi and bacteria. Though, most of the traditionally fermented food and beverages are dominated by the yeast *Saccharomyces cerevisiae,* and to a lesser extent *Lactobacillus* (Jespersen 2003; Ogbulie et al. 2007; Karamoko et al. 2012; Rokosu and Nwisienyi 1980), the presence of other microbes can lead to the production of diverse products including methanol (Dato et al. 2005; Shale et al. 2013; Kostik et al. 2014). Several compounds could be produced during mixed fermentation with several organisms. Also, it has been severally reported that microbial fermentation of substrates rich in pectin can result in the formation of methanol (Nakagawa et al. 2000; Mendonca et al. 2011; Siragusa et al. 1988). Contaminating yeast has been demonstrated to produce methanol during traditional fermentation (Dato et al. 2005). Recent studies have also shown that the ethanol fermenting yeast, *S. cerevisiae* has several strains with slightly different metabolism (Jespersen 2003; Stringini et al. 2009; Okunowo et al. 2005) with some strains possibly producing methanol. More worrisome are recent studies showing increase in blood methanol level in some persons even after consumption of methanol-free ethanol (Shindyapina et al. 2014; Dorokhov et al. 2015). These authors recognized two sources of methanol in human systems, endogenous and exogenous sources. It is generally believed that unscrupulous vendors deliberately spike beverages with methanol in order to increase the alcohol content. The aim of this review is to present alternative viewpoint showing the possible role of microbes in the production of methanol in traditionally fermented beverages. We reviewed literatures on traditionally fermented alcoholic beverages, assessed the methanol content of the beverages, the pectin content of their feedstocks and the microbial species involved in the fermentation in an attempt to establish a possible role of microbes in the production of methanol in traditionally fermented alcoholic beverages.

## Methanol contamination in fermented beverages

The result of the review is presented in Table 1, showing that several traditionally fermented alcoholic beverages in different countries could be prone to methanol contamination. Majority of the beverages are made from few feedstocks including palm wine, sorghum, millet, maize, sugarcane, citrus, banana, milk and Plum. Cases of methanol contamination have been reported in some of the wines produced from banana, plum and Agrave. Spirits made from mangoes, pears, banana and melon have been shown to contain methanol (Mendonca et al. 2011). In Rwanda, traces of methanol were reported in Urwagwa, a beer produced from banana (Shale et al. 2013). Mendonca et al. (2011) reported 0.05–0.189 % methanol in cachaca produced from banana pulp, while Dato et al. (2005) reported 0.00–0.50 % methanol in cachaica produced from sugarcane in Brazil. Plum wine (Joshi et al. 2009; Jung et al. 2010), plum brandy (Kostik et al. 2014), agave (Leon-Rodriguez et al. 2008) contain methanol. The substrate for ethanol production is the first probable source of methanol in the beverage. Chaiyasut et al. (2013) reported factors affecting the methanol production in fermented beverages including raw material size and age, sterilization temperature, pectin content and pectin methyl esterase (PME) activity (Note that PME activity is optimal at 50–60 °C).

**Table 1:** Traditionally fermented alcoholic beverages prone to methanol contamination

Beverage	Feedstock	Fermenting organism	Countries	Alcohol content	References
Palm wine	silver date palm (*Phoenix sylvestris*), the palmyra, jaggery palm (*Caryota urens*), oil palm (*Elaeis guineense*) *Raffia palms*, *kithul* palms, or *nipa* palms. coconut palms *Borassus*	Yeast (*Saccharomyces cerevisiae, Saccharomyces ludwigii*, *Candida parapsilosis, Candida fermentati, Pichia fermentans, Schizosaccharomyces pombe, Schizosaccharomyces bailli, Kluvyeromyces africanus, Hansenula auvarum, Kloeckera apiculata, Torulaspora delbrueckii*) & Lactic Acid Bacteria (*Lactobacillus*, *Leuconostoc*, *Pediococcus*, *Lactococcus*, and *Streptococcus*), acetic acid bacteria (*Acetobacter, Aerobacter*)	Most African and Asian countries		Ogbulie et al. (2007), Rokosu and Nwisienyi (1980) and Karamoko et al. (2012)
Local gin (ogogoro, kaikai, apetesi)	Palm wine	(*Saccharomyces cerevisiae*) & bacteria (*Lactobacillus*)	Most African and Asian countries	40–60 % Ethanol	Ohimain et al. (2012)
Pito (local beer)	Sorghum or maize	Bacteria (*Pediococcus halophilus, Lactobacillus*) & yeast (*Saccharomyces cerevisiae, Candida tropicalis, Schizosaccharomyces pombe, Kluvyeromyces africanus, Hansenula anomala, Kloeckera apiculata, Torulaspora delbrueckii*)	West Africa	2–3 % Ethanol	Orji et al. (2003), Sefa-Dedeh et al. (1999) and Iwuoha and Eke (1996)
Burukutu	Sorghum	*Sacharomyces cerevisiae, Streptococcus, Lactobacillus, Aspegillus, Fusarium, Penicillium*	Nigeria, Ghana	1.63 % ethanol	Eze et al. (2011) and Iwuoha and Eke (1996)
Tchapalo (sorghum beer)	Sorghum	Lactic acid bacteria	Cote d’Ivoire		Aka et al. (2008)
Tchapalo (sorghum beer)	Sorghum	Lactic acid bacteria (several species)	Cote d’Ivoire		Koffi-Marcellin et al. (2009)
Bushera	Sorghum	Lactic acid bacteria (several species)	Uganda	0.20–0.75 % ethanol	Muyanja et al. (2003)
Ogi	Maize, sorghum or millet	*Sacharomyces cerevisiae, Lactobacillus plantarum, Streptococcus lactis*	Nigeria	?	Iwuoha and Eke (1996)
Urwagwa (banana beer)	Banana		Rwanda	8.7–18 (ethanol), trace (methanol)	Shale et al. (2013)
Cachaca (banana pulp wine)	Banana	*Sacharomyces cerevisiae*	Brazil	Ethanol (5.34–7.84 %), methanol (0.65–0.189 %)	Mendonca et al. (2011)
Cachaca	Sugarcane	*Sacharomyces cerevisiae* and wild yeasts (*Pichia* sp & *Dekkera bruxelensis*)	Brazil	Methanol (0–0.5 %)	Dato et al. (2005)
Noni	Morinda trifolia	*Lactobacillus plantarum & L. casei*	Thailand	853 mg/l methanol	Chaiyasut et al. (2013)
Kwunu-zaki	Millet	*Sacharomyces cerevisiae*	Nigeria	?	Iwuoha and Eke (1996)
Cocoa sap wine	Cocoa sap	*Sacharomyces cerevisiae*	Nigeria	?	Iwuoha and Eke (1996)
Cholai	rice, sugar-cane, juice of date tree, molasses, and fruit juice (pineapple and jackfruits)	*Sacharomyces cerevisiae*	India	14.5 % alcohol	Islam et al. (2014)
Dengue	Millet	Lactic acid bacteria (several species)	Burkina Faso		Quattara et al. (2015)
Yoghurt	Milk	Lactic acid bacteria (several species)	Iran		Azadnia and Khan (2009)
Gariss	Milk	Lactic acid bacteria (several species)	Sudan		Ashmaig et al. (2009)
Kwete	Maize & millet	Lactic acid bacteria	Uganda		Namuguraya and Muyanja (2009)
Agave	Agave		Mexico	3.9–339 g/l (ethanol), ND-1826 mg/l (methanol)	Leon-Rodriguez et al. (2008)
Plum wine	Plum		Romania	53–76 % (ethanol), 554–4170 mg/l (ethanol)	Jung et al. (2010)
Plum brandy	Plum		Macedonia	47–51 % (ethanol), 564–999 mg/l (methanol)	Kostik et al. (2014)
Plum wine	Japanese Plum (*Prunus salicina* Linn)	Yeast	India	175 mg/l Methanol	Joshi et al. (2009)

Another possible source of methanol in traditionally fermented alcoholic beverage is the fermenting microbes. The ethanol fermenting yeast *S. cerevisiae* dominated traditional fermentation followed by *Lactobacillus* (Table 1). Jespersen (2003) also observed this trend in African indigenous fermented beverages and foods. *Saccharomyces cerevisiae* have been used as catalysts for the production of ethanol for thousands of years. But recent studies have shown that there are different strains of *S. cerevisiae* involved in traditional ethanol fermentation (Hayford and Jespersen 1999; Jespersen 2003; Kuhle et al. 2001; Pataro et al. 2000; Guerra et al. 2001; Ezeronye and Legras 2009). The big question is ‘have the traditional ethanol producing yeast evolved into the production of methanol in addition’? Professor Benito Santiago, University of Spain (Personal communication, July 2015) opined that some years ago, methanol at low concentration was desirable in beer and wines. However, we were unable to find literature confirming this claim.

Pectins are a group of heterogeneous polysaccharides found in the intercellular regions and cell walls of most fruits and vegetables (Siragusa et al. 1988), with its greatest abundance in citrus particularly orange, grape, limes and lemons (Siragusa et al. 1988). Citrus contains 7–10 % pectin (Siragusa et al. 1988). Chaiyasut et al. (2013) compared pectin levels in fermented beverage containing *Morinda citrifolia* (9.89 %) with that of other fruits including guava (4.36 %), tomato (0.3 %), apple (0.5 %), carrot (0.8 %) and cherries (0.4 %). During ripening, pectin in fruits is broken down by PME resulting in the formation of methanol (Chaiyasut et al. 2013; Micheli 2001). However, pectin has not been reported in palm wine.

Plant cell wall degrading enzymes including pectinases are ubiquitous among pathogenic and saprophytic bacteria and fungi (Prade et al. 1999). Pectin enzymes are widely distributed in nature and are produced by yeast, bacteria, fungi and plants (Sieiro et al. 2012). Methanol is a major end product of pectin metabolism by microorganisms (Schink and Zeikus 1980). Human colonic bacteria, *Erwinia carotovora* is able to degrade pectin releasing methanol (Siragusa et al. 1988). Anaerobic bacteria, particularly *Clostridium butyricum*, *Clostridium thermocellum*, *Clostridium multifermentans*, and *Clostridium felsineum* produce methanol from pectin (Ollivier and Garcia 1990). Schink and Zeikus (1980) reported various pectinolytic strains of *Clostridium*, *Erwinia* and *Pseudomonas*. Dorokhov et al. (2015) listed at least 20 species of human colonic microbes capable of producing methanol endogenously. The authors in a comprehensive review presented at least five different pathways of methanol synthesis in humans and four pathways of methanol clearance from the body and they also demonstrated the presence of gene regulation in methanol synthesis. Readers are advised to consult this literature for details on metabolic methanol in human systems.

Pectinolytic enzymes are classified into esterases and depolymerase (lyases and hydrolases). Hydrolysis of pectin by lyases produces oligo- or mono-galacturonate, while hydrolysis of pectin by esterases produces pectic acid and methanol (Sieiro et al. 2012). Some authors have identified strains of *Saccharomyces* that produces the three types of pectinolytic enzymes namely pectin methyl esterase (PME, EC: 3.1.1.11), pectin lyase (PL), and polygalacturonase (PG) (Gainvors et al. 1994a, b; Naumov et al. 2001). Fernandez-Gonzalez et al. (2005) genetically modified *S. cerevisiae* strain having pectinolytic activity. Analysis of *S. cerevisiae* among many traditional fermented beverages in Africa shows that they vary according to the location and types of substrates (Jespersen 2003). Strains of *S. cerevisiae* having PME activity could produce methanol during fermentation.

Methanol is produced during fermentation by the hydrolysis of naturally occurring pectin in the wort (Nakagawa et al. 2000; Mendonca et al. 2011). PME de-esterify pectin to low—methoxyl pectins resulting in the production of methanol (Chaiyasut et al. 2013; Micheli 2001).

Jespersen (2003) reported the roles of *S. cerevisiae* in the traditional fermentation to include fermentation of carbohydrate to ethanol, production of aromatic and flavor compounds, stimulation of lactic acid bacteria and probiotic activities among others. *Saccharomyces cerevisiae* also inhibit the mycotoxin producing fungi and cause the degradation of poisonous cyanogenic glycosides and produces tissues degrading enzymes such as cellulose and pectinase. The volume of ethanol produced during fermentation is dependent on the strains of yeast used. For instance, the total alcohol (ethanol and methanol) produced from orange juice fermentation was 3.19 % w/v with *S. cerevisiae var. ellipsoideus* and 6.80 % w/v with *S. carlsbergensis* (Okunowo and Osuntoki 2007). During the production of sugarcane beverage called cachaca in Brazil, *S. cerevisiae* produced no methanol while contaminating yeasts (*Pichia silvicola* and *P. anomala*) produced 0.5 % methanol (Dato et al. 2005). Stringini et al. (2009) studied yeast diversity during tapping and fermentation of oil palm wine from Cameroon and found *S. cerevisiae*, *Saccharomyces ludwigii*, *Schizosaccharomyces bailli*, *Candida parapsilosis*, *Pichia fermentans*, *Hanseniaspora uvarum* and *Candida fermentati* in addition to lactic acid bacteria and acetic acid bacteria. Literature abounds on the microbiology of traditionally fermented beverages. Karamoko et al. (2012) isolated yeasts, mould and bacteria including *Bacillus*, *Brevibacterium*, *Micrococcus* and *Escherichia coli*. Rokusu and Nwisienyi (1980) isolated lactic acid bacteria (*Lactobacillus*, *Streptococcus* and *Leuconostoc*) and Acetic acid bacteria (*Acetobacter* and *Aerobacter*). Stringini et al. (2009) using molecular techniques reported the diversity of yeasts involved in palm wine fermentation including *S. cerevisiae* and other yeast such as *Candida parapsilosis, C. fermentati* and *Pi*c*hia fermentans*. Similarly, the microbiology of other traditionally fermented alcoholic beverages and foods have been well documented (Ogbadu et al. 1997; Muyanja et al. 2003; Namuguraya and Muyanja 2009; Quattara et al. 2015; Koffi-Marcellin et al. 2009; Ashmaig et al. 2009; Eze et al. 2011).

Since traditional fermentation occur via spontaneous inoculation from the substrate and processing equipment (Ohimain et al. 2012; Jespersen 2003), hence mixed cultures usually carry out the fermentation. Therefore, contaminating microbes including other yeasts, fungi and bacteria could result in the production of several other products including methanol. And because methanol has a lower boiling point (65 °C) than ethanol (78 °C), it could be further concentrated in the beverage during distillation. Though, there are some disadvantages of mixed culture fermentation, the use of mixed culture in ethanol production will offer the advantage of production at low cost since a large range of substrates may be metabolized into ethanol. Moreover, the high cost associated with operations of process plants with pure cultures could be drastically minimized when mixed cultures are used.

As previously stated, mixed fermentation could result in the production of diverse products. Even pure culture fermentation can result in the production of diverse products depending on the operating conditions. Hence, beverages produced via spontaneous fermentation by mixed culture could produce greater variety of products. Table 2 listed some volatile congeners produced in selected alcoholic beverages beside methanol. Some of these compounds are also very poisonous e.g. ethyl carbamate and some are even carcinogenic (Lachenmeier et al. 2009, 2011; Testino et al. 2014; Testino and Borro 2010). Annan et al. (2003) listed 64 volatile compounds produced during the mixed culture fermentation of Ghanaian maize dough consisting of 20 alcohols, 22 carbonyls, 11 esters, 7 acids, 3 phenolic compounds and a furan.

**Table 2:** Some volatile congeners in alcoholic beverages

Congener	Concentration	Beverages	References
1-butanol	ND to 35 mg/l	Agrave	Leon-Rodriquez et al. (2008)
	8 to 74 mg/l	Plum wine	Jung et al. (2010)
	4.5 to 12 mg/100 ml	Plum brandy	Kostik et al. (2014)
	1.0 to 5.2 mg/100 ml	Grape brandy	Kostik et al. (2014)
	ND to 9.8 mg/l	Raki	Gueven (2013)
2-butanol	ND to 59 mg/l	Agrave	Leon-Rodriquez et al. (2008)
	309 to 1092 mg/l	Plum wine	Jung et al. (2010)
	14.5 to 55 mg/100 ml	Plum brandy	Kostik et al. (2014)
	1.5 to 110.5 mg/100 ml	Grape brandy	Kostik et al. (2014)
	ND to 18.39 mg/l	Raki	Gueven (2013)
1-propanol	ND to 708 mg/l	Agrave	Leon-Rodriquez et al. (2008)
	76 to 1141 mg/l	Plum wine	Jung et al. (2010)
	22 to 305 mg/100 ml	Plum brandy	Kostik et al. (2014)
	4.1 to 90.5 mg/100 ml	Grape brandy	Kostik et al. (2014)
	ND to 727 mg/l	Raki	Gueven (2013)
2-propanol	12.2 to 26.5 mg/100 ml	Plum brandy	Kostik et al. (2014)
	7 to 26.5 mg/100 ml	Grape brandy	Kostik et al. (2014)
Acetic acid	ND to 1192 mg/l	Agrave	Leon-Rodriquez et al. (2008)
Acetone	25 to 40 mg/l	Plum wine	Jung et al. (2010)
Aldehyde	ND to 67.3 mg/l	Raki	Gueven (2013)
Ethyl acetate	ND to 30.19 mg/l	Wines and spirits	Osobamiro (2013)
	100 to 474 mg/l	Agrave	Leon-Rodriquez et al. (2008)
	48 to 454 mg/100 ml	Plum brandy	Kostik et al. (2014)
	5.2 to 255 mg/100 ml	Grape brandy	Kostik et al. (2014)
	12.8 to 292 mg/l	Raki	Gueven (2013)
Ethyl carbamate	378 to 421 µg/kg	Yellow rice wine	Wu et al. (2014)
	ND to 40.65 mg/l	Wines and spirits	Osobamiro (2013)
	<0.15 mg/l	Agrave	Lachenmeier et al. (2009)
Higher alcohol^a^	267 to 2007 mg/l	Agrave	Leon-Rodriquez et al. (2008)
	ND to 2275 mg/l	Raki	Gueven (2013)

^a^Higher alcohol are alcohols with molecular weight higher than ethanol i.e. alcohol that has more than 2 carbon; *ND* not detected

## Recommendations and the way forward

Paine and Davan (2001) reported that low concentrations of methanol occur naturally in most alcoholic beverages without causing any harm. According to WHO (2014), methanol concentration of 6–27 mg/l in beer and 10–220 mg/l in spirits are not harmful. Paine and Davan (2001) reported that the daily safe dose of methanol in an adult is 2 g and a toxic dose of 8 g as against the EU general limit for naturally occurring methanol of 10 g methanol/ethanol, which is equivalent to 0.4 % (v/v) methanol at 40 % ethanol. Czech Republic permitted safe limit for methanol in spirits is 12 g/l of pure ethanol (Vaskova 2013). Note that EU Methanol limit is variable (0.2–1.5 %) depending on the type of beverage and feedstock used for fermentation. Some countries have regulatory limits of methanol in alcoholic beverages (Table 3). This regulatory control should be encouraged rather than outright ban.

**Table 3:** Regulatory limits of methanol in beverages

Country	Maximum methanol value^a^	Reference
Brazil	0.5 % (0.5 ml/100 ml)	Mendonca et al. (2011)
Thailand	0.024 % (240 mg/l)	Chaiyasut et al. (2013)
Australia/New Zealand	0.8 % (8 g/l)	Chaiyasut et al. (2013)
EU^b^	200 g/hl (0.2 % for wine & brandy, 1000 g/hl (1 %) for grape marc spirit and fruit spirit, 1500 g/hl (1.5 %) for fruit marc spirit	European Community (2008)
USA	0.1 %	FDA (Federal Food, Drug and Cosmetic Act 21 USC 34 (a)(2)(C)
Vietnam	0.3 %	Socialist Republic of Vietnam (2010)
Nigeria	0.0005 % (5 mg/l)	NAFDAC (2005)

^a^Concentration of methanol in ethanol

^b^EU limits for methanol in alcoholic beverages is variable depending on the type of beverage and the feedstock used for fermentation

Microbiological control of the process could also be used to prevent methanol formation in fermented beverages. For instance, pure culture inoculation using commercial yeast as opposed to spontaneous inoculation by wild yeasts should be practiced. The traditional fermentation processes could also be scaled-up using well characterized and purified starter culture. For instance, starter cultures have been successfully used to produce pito, a traditionally fermented alcoholic beverage produced from maize or sorghum (Orji et al. 2003). Adequate equipment with process controls should be used for fermentation and distillation as opposed to rudimentary equipment lacking controls, which are currently used. For instance, sterilization/boiling at temperatures higher than 80 °C could prevent the production of methanol (Chaiyasut et al. 2013; Amaral et al. 2005). Moreover, standard microbiological process controls and working under aseptic conditions could control contaminating wild yeasts in the fermentation process. Jespersen (2003) also recommended improved process control of fermentation and product characterization including the use of purified starter cultures with appropriate technology.

Another microbiological method for the control of methanol in fermented beverages, is the use of methylotrophic yeast such as *Pichia methanolica* (Nakagawa et al. 2005) and *Candida boidinii* (Nakagawa et al. 2000) which have the capacity of utilizing pectin or methyl ester moiety of pectin and methanol, thus preventing the accumulation of methanol in fermented products. Finally, instead of an outright ban on traditional fermentation, because of methanol contamination, the mixed alcohol (ethanol and methanol) could be further processed and used as biofuel. Literature abounds on the use of methanol and ethanol as biofuels (Kamboj and Karimi 2014; Iliev 2015; Shayan et al. 2011).

## Conclusions

Incidences of methanol contamination in traditional beverages are increasing globally and have caused death in many counties including Nigeria, India and Indonesia. It is generally believed that unscrupulous vendors deliberately spike the beverages with methanol in order to increase the alcohol content. This review observed that methanol production in traditional fermented beverages can be linked to the activities of pectinase producing yeast, fungi and bacteria. Microbes producing pectin methyl esterase are able to produce methanol from fruits/juices containing pectin. Under traditional/informal fermentation, alcoholic beverages produced by mixed microbial consortium could probably lead to the production of mixed alcohols containing methanol and other volatile congeners. The study concluded by suggesting that contaminated alcoholic beverages be converted for fuel use rather than out rightly banning the age—long traditional alcohol fermentation. Regulatory limits for methanol in fermented beverages should be strictly enforced. It is also suggested that pure cultures should be used for alcohol fermentation under aseptic conditions as opposed to spontaneous fermentation by mixed contaminating microbes.
